# Financial and relational impact of having a boy with posterior urethral valves

**DOI:** 10.3389/fped.2023.1228248

**Published:** 2023-08-09

**Authors:** Luke Harper, Nathalie Botto, Matthieu Peycelon, Jean-Luc Michel, Marc-David Leclair, Sarah Garnier, Pauline Clermidi, Alexis Arnaud, Anne Dariel, Eric Dobremez, Alice Faure, Laurent Fourcade, Nadia Boudaoud, Yann Chaussy, Laetitia Huiart, Valery Bocquet, Cyril Ferdynus, Frédérique Sauvat

**Affiliations:** ^1^ Department of Pediatric Surgery, CHU de La Réunion, Saint Denis de La Réunion, France; ^2^ Department of Pediatric Surgery and Urology, University Hospital Pellegrin-Enfants, CHU de Bordeaux, Bordeaux, France; ^3^ Department of Pediatric Surgery and Urology, APHP, Hôpital Necker, Paris, France; ^4^ Department of Pediatric Urology, University Hospital Robert Debre, APHP, University of Paris, Centre de Référence des Malformations Rares des Voies Urinaires (MARVU), Paris, France; ^5^ Department of Pediatric Surgery and Urology, Children’s University Hospital, CHU de Nantes, Nantes, France; ^6^ Department of Pediatric Surgery and Urology, Lapeyronie University Hospital, CHU de Montpellier, Montpellier, France; ^7^ Department of Pediatric Surgery, Armand Trousseau Children’s University Hospital, Paris, France; ^8^ Department of Pediatric Surgery, Rennes University Hospital, CHU de Rennes, Rennes, France; ^9^ Department of Pediatric Surgery, North and Timone Children’s Hospital, Assistance Publique Hopitaux de Marseille, Aix-Marseille Université, Marseille, France; ^10^ Department of Pediatric Surgery, University Hospital, CHU de Limoges, Limoges, France; ^11^ Department of Pediatric Surgery, Reims University Hospital, Reims, France; ^12^ Department of Pediatric Surgery, Besançon University Hospital, CHU de Besançon, Besançon, France; ^13^ Unité de Soutien Méthodologique, CHU de La Réunion, Saint-Denis de La Réunion, France; ^14^ Clinical Research Department, INSERM, CIC1410, CHU de la Réunion, Saint-Pierre, France

**Keywords:** posterior urethral valves, impact, family, global healthcare, chronic disease

## Abstract

**Introduction:**

Childhood chronic diseases affect family functioning and well-being. The aim of this study was to measure the impact of caring for a child with PUV, and the factors that most impact the burden of care.

**Patients and method:**

We gave a questionnaire on the familial impact of having a child with posterior urethral valves to all parents of a child included in the CIRCUP trial from 2015 onwards. The questionnaire included questions about the parents' demographics, health, professional, financial and marital status and how these evolved since the child's birth as well as the “impact on family scale” (IOFS), which gives a total score ranging from 15 (no impact) to 60 (maximum impact). We then analyzed both the results of the specific demographic questions as well as the factors which influenced the IOFS score.

**Results:**

We retrieved answers for 38/51 families (74.5% response rate). The average IOFS score was 23.7 (15–51). We observed that the child's creatinine level had an effect on the IOFS score (*p* = 0.02), as did the parent's gender (*p* = 0.008), health status (*p* = 0.015), being limited in activity since the birth of the child (*p* = 0.020), being penalized in one's job (*p* = 0.009), being supported in one's job (*p* = 0.002), and decreased income (*p* = 0.004). Out of 38 mother/father binomials, 8/33 (24.2%) declared that they were no longer in the same relationship afterwards.

**Conclusion:**

In conclusion, having a boy with PUV significantly impacts families. The risk of parental separation and decrease in revenue is significant. Strategies aiming to decrease these factors should be put in place as soon as possible.

## Introduction

Childhood chronic diseases affect family functioning and well-being. Parents need to assume a complex role, both serving as parent and care coordinator, and these adaptations can alter parental health-related quality of life (HRQoL). Assessing family impact of chronic childhood illness is useful to identify the need for family education, psychological intervention, and social support. This assessment is important both for health care professionals and policy makers to improve the HRQoL of children and their caregivers (1).

Posterior urethral valves (PUV) are obstructing membranous folds within the lumen of the posterior urethra forming the most common cause of Lower Urinary Tract Obstruction (LUTO) in boys. They affect 1:4,000 to 1:25.000 births, and cause increased intravesical pressure during fetal kidney development resulting in various degrees of kidney and bladder impairment (2). The clinical spectrum of PUV is wide thus contributing to prognostic uncertainty and singular clinical trajectories, but all these children present a certain degree of kidney and bladder impairment and will require close and repeated medical attention during childhood.

Most studies that explore the burden of care for a child with kidney disease have focused primarily on dialysis and transplant patients, though a recent paper did study the families of PUV patients (3–6). The aim of this study was to measure the impact of caring for a child with PUV, and more specifically the factors, related to the child or the parents, that most impact the burden of care. We hypothesized that the impact on family depends both on the severity of the child's disease but also on family socio-economic factors.

## Patients and method

We conducted the *IFUP* study (study acronym—full french title: **I**mpact sur les **F**amilles d'avoir un garçon porteur de valves de l'**U**rètre **P**ostérieur) by giving a questionnaire on the familial impact of having a child with posterior urethral valves to the parents of children who were participating in the *CIRCUP* randomized controlled trial. The *CIRCUP* trial (Clinicaltrials registration number: NCT01537601) was a multicentric study which began in 2012, and included 92 boys with PUV, the aim of which was to determine whether circumcision decreased the risk of febrile UTI in boys with PUV (7). The *IFUP* study was added to the CIRCUP trial in 2015, meaning that a specific consent form was added to the CIRCUP consent form, and the questionnaire was offered to all parents of a child included in the CIRCUP trial from 2015 onwards. Inclusion ended in July 2017 as the number of required participants for the CIRCUP trail was reached. The *IFUP* study received formal ethics committee approval.

Each parent filled a questionnaire, which included questions about demographics as well as their health, professional, financial and marital status and how these evolved since the child's birth. We included the “impact on family scale” (IOFS), a validated parent-reported measure of familial impact (8). This instrument, initially developed by Stein et al, has been progressively reduced to a questionnaire comprising 15 items with a main dimension representing the general negative impact on social and family spheres. This instrument had been previously validated in French (9). The questionnaire was given to the parents at the last visit of the CIRCUP protocol which took place when the child was two years of age. Parents either filled it in immediately or send it back by postal mail after completing it at home. The total impact score was calculated by summing the results of all items, giving a total score ranging from 15 (no impact) to 60 (maximum impact).

Demographic characteristics were described by their frequency and percentages for categorical variables and by means and standard deviations or medians and interquartile ranges for quantitative variables. Cohen's f2 (10) was used as a measure of effect size to quantify the magnitude of the relationship between the following factors and the IOFS score: being penalized in their job, having activities limited since the child's birth, decrease in income, state of health, being supported in their job, impact on their health, severity of the child's disease (creatinine as proxy), having family support, having psychological support. Cohen's f2 was estimated using a linear mixed model with the parent type (i.e., mother or father) as a random effect to take intra family correlation into account. The Bland-Altman plot was used to analyze the agreement between mothers and fathers responses. All statistical tests for were performed at a 2-tailed type I error of 5%. All analyses were performed used SAS version 9.4 (SAS Institute, Cary, North Carolina).

## Results

Out of the 92 children included in the *CIRCUP* study, 41 had finished their follow-up at the beginning of the *IFUP* study. Thus, the questionnaire was offered to 51 families of which 38 answered (74.5% response rate). In 28 cases we had answers from both the mother and the father, in 10 cases we had only the mother's answers and in one case only the father's, for a total of 67 completed questionnaires. The average age of the respondents was 34.9 (6.1) years.

The average IOFS score was 23.7 (8.0), with a median score of 22.0 (17-30) and a range of 15 to 51. The average score for mothers was 24.7 (8.9) (median:24.0 (18.0–30.0), and the average score for fathers was: 22.4 (6.7) (median: 22.0 (15.0–27.0). Agreement between parents was good with homogenous distribution around the mean. The main results are presented in Table 1.

**Table 1:** Main results parent questionnaire.

	Total
Age, mean (SD)	34.9 (6.1)
Female, *n* (%)	37 (56.1%)
Marital status, *n* (%)	
Single	14 (21.2%)
Divorced	6 (9.1%)
Living as a couple	25 (37.9%)
Married	21 (31.8%)
Number of children, mean (SD)	2.7 (1.2)
Was your state of health influenced, *n* (%)	
Yes, a lot	2 (3.1%)
Yes, a little	18 (28.1%)
No, not really	14 (21.9%)
No, not at all	30 (46.9%)
Activities limited within the past 2 years, *n* (%)	
Totally agree	7 (10.8%)
Somewhat agree	10 (15.4%)
Neither agree nor disagree	6 (9.2%)
Somewhat disagree	10 (15.4%)
Totally disagree	32 (49.2%)
Penalized in your job, *n* (%)	
Yes, by my employer	3 (4.8%)
Yes, by my colleagues	0 (0.0%)
Yes, by my employer and my colleagues	1 (1.6%)
No, not at all	58 (93.6%)
Supported in your job, *n* (%)	
Yes, by my employer	3 (5.0%)
Yes, by my colleagues	10 (16.7%)
Yes, by my employer and my colleagues	20 (33.3%)
No, not at all	27 (45.0%)
Did you have to stop working? *n* (%)	
No	38 (62.3%)
Yes	23 (37.7%)
How long? *n* (%)	
Less than 2 weeks	14 (63.6%)
Less than 2 months	3 (13.6%)
Less than 6 months	1 (4.5%)
Between 6 months and a year	0 (0.0%)
More than a year	4 (18.2%)
Decrease in revenue, *n* (%)	
Yes, a lot	3 (4.7%)
Yes, a little	10 (15.6%)
No, not at all	51 (79.7%)
Living as a couple, *n* (%)	
No	16 (24.6%)
Yes	49 (75.4%)
Living as a couple before your child's diagnosis? *n* (%)	
No	6 (9.2%)
Yes	59 (90.8%)
Living as a couple with the same person? *n* (%)	
No	18 (29.0%)
Yes	44 (71.0%)
Psychological support *n* (%)	
No	62 (95.4%)
Yes	3 (4.6%)
Helped in coping with your child? *n* (%)	
No, never	19 (29.7%)
Yes, sometimes	25 (39.1%)
Yes, often	15 (23.4%)
Yes, daily	5 (7.8%)

The most remarkable points are that the parent's state of health was influenced by the child's condition in 31% of cases, and the parents' activities were limited in 26% of cases. 20% suffered from a loss of revenue and 38% had to leave work, of which 18% had to for more than a year. Out of 38 mother/father binomials, 33 (86.8%) declared that they were living as a couple when the child was born, but 8 declared that they were no longer in the same relationship afterwards, i.e., a proportion of separation of 24.2% (IC95%: 9.6%–38.9%).

Though the IOFS score does increase as the child's creatinine increases (an increase in nadir creatinine of 10 µmol/L is associated with an increase of 0.7 in the IOFS score), Cohen's f2 showed that the main modifiers of the IOFS score are related to the parents. They are limitation in activity and being penalized professionally (Table 2).

**Table 2:** Effect size of each variable on IOFS score.

Variables	Cohen's f2	Effect size Interpretation
Penalized in your job	0.88*	Large
Activities limited since your child's birth	0.75*	Large
Decrease in income	0.29	Medium
State of health	0.29	Medium
Supported in your job	0.26	Medium
Impact on your health	0.25	Medium
Disease severity (creatinine as proxy)	0.19	Medium
Family support	0.10	Low
Psychological support	0.09	Low

Cohen's f2 for local effect sizes adjusted for role of parent (father or mother) and interaction with variable of interest.

* Significant interaction between parent and variable of interest..

## Discussion

Having a boy with PUV impacts the family in many ways. Previous reports suggest that impact varies with severity of the condition with mean scores reaching 36–42 in cases of chronic respiratory failure, orphan/rare diseases or palliative care (11–13). It is generally believed that IOFS scores <30 indicated a low level of impact, between 30 and 45 indicated significant impact, and >46 indicated very serious impact. In our study, the average score was 23.7 which is very close to the score observed in the study by Tan et al. on the same population (score of 23). This seemingly low score could be interpreted as meaning that the impact on families is relatively low (6), though 12 parents (18.2%) showed a score >30 and 2 (3.0%) a score >45. But in reality, more than the average IOFS score which expresses how families, on average, cope with having a child with PUV, the true interest of these scales lies in the factors which impact it.

There was one factor related to the child which impacted impact on family and that was the nadir creatinine. This is understandable as we used nadir creatinine as a proxy for disease severity and it seems intuitive that the more severe the disease is, the more the situation impacts the family. However, using Cohen's f2, disease severity had a medium size impact on the IOFS score. The factors with the highest impacts were related to the parents. The two main modifiers were: being limited in activities since the child's birth and being penalized professionally. Having support whether familial or psychological, impacted relatively less than the other factors.

The previous study by Tan et al, which is cross-sectional, included children of various ages and from a single center and looked at the impact on IOFS of demographic factors, including where the family lived as well as whether they viewed their care as family-centered. Our study is multicentric, uses the same time-point for all children (2 years), and aimed to capture how the parents' health, financial and marital status evolved during the child's first two years of life. This brought us data, which were not available from previous studies. The result that surprised us most was the impact on marital status. According to our national statistics, the separation rate is 1.1% per year and in this study it was 24% over 2 years which is a 10-fold increase (14). It is true that the national statistics only takes into account marriages and divorces, and that we don't have the true separation rate in the general population, but this confirms previous reports that have shown that having a child with a chronic disease puts a significant burden on the parents' relationship. In the study by Reichman et al. (15), they found that within 12 to 18 months after the birth, having a child with poor health decreased the likelihood that the parents were living together by 10%. The strong effect of children's poor health on parents' relationship status, for those who cohabited at baseline, whether married or not, indicated that previous studies that focused on marriage and divorce told only part of the story.

Recognition of the extent and nature of the impact of chronic illness on families is crucial to improve patient and family-related outcomes. The question that does arise is how the information on family burden of care should be presented during prenatal counselling. Preparing and informing parents is the main goal of prenatal counselling but it might be difficult to both discuss the child's renal and bladder prognosis as well as the risk for the parents to lose revenue and risk separation before the child is born. It is important for health care professionals to know this so that they can try to encourage parents to get all the available help as soon as possible.

The main limitations of our study are related to the total number of parents who answered the questionnaire; PUV is a relatively rare disease but our numbers don't allow for subgroup analysis according for instance to initial family income, socio-economic status, level of education, age of the parents, number of siblings, etc .…, though obviously all these factors can impact coping. Also, the fact that we did not always have both parents can induce a form of bias, as both parents don't experience things exactly the same way. Furthermore, our study reflects only the first two years of life, and the impact on families can evolve as the disease evolves with time, for better or worse. On the other hand, our strength includes the fact that all measures were taken at the same time frame for each family and that the children came from multiple institutions and many different backgrounds and reflects the global situation of parents coping with a child with PUV.

In conclusion, having a boy with posterior urethral valves significantly impacts families. The risk of parental separation and decrease in revenue is significant. Strategies aiming to decrease these factors should be put in place a soon as possible.

## Data Availability

The raw data supporting the conclusions of this article will be made available by the authors, without undue reservation.
